# Disseminated Knowledge: The Advancement of Finnish Occupational Medicine and Work Psychology in a Transnational Context, c. 1945–1952

**DOI:** 10.1093/shm/hkae067

**Published:** 2024-11-12

**Authors:** Mona Mannevuo

**Affiliations:** Helsinki Collegium for Advanced Studies, University of Helsinki, Fabianinkatu 24 A, 00014, Helsinki, Finland

**Keywords:** Finland, industrial neurosis, mental hygiene, Roffey Park, work psychology

## Abstract

This article focuses on the advancement of Finnish occupational medicine in the immediate post-war period, situating its development within a transnational context. Its objective is to offer insight into Finnish post-war industrial medicine and particularly developments in mental health care. The empirical methodology addresses a previously unexplored case study: the connections between the Finnish Institute of Occupational Health (FIOH) and Roffey Park Rehabilitation Centre, established in 1943 to address various cases of industrial neurosis. The case study sheds light on the ways in which FIOH adopted reformist ideas from transnational medical communities by aligning them with the needs of Finland’s war reparations industry. The article argues that FIOH’s experts advanced new theories of mental disorder for Finland’s newly modern industrial society, and that these initiatives should be situated within broader transnational endeavours in the mental hygiene movement.

‘In recent years, experts in occupational medicine have become more certain that many diseases originate from poor social conditions, mental hardships, and environmental factors’, asserted physician Erkki Leppo (1909–96) in a study trip report written for the Finnish Institute of Occupational Health (FIOH) in 1948.^1^ Leppo belonged to a new generation of Finnish physicians who were exceptionally well integrated into the English-speaking medical communities. This study trip to the UK, funded by the Rockefeller Foundation, exemplifies how the recently established FIOH reformists were actively discovering new ways to understand work-related illnesses, absenteeism and maladaptation to modern industrial life.

The aim of the excursion was to learn how medical services were organised in the UK to initiate occupational health reform in post-war Finland. Compared to many other European countries, Finland industrialised relatively late. For a large proportion of the population, until the end of the 1950s, the chief source of livelihood was agriculture. In 1945, only 25 per cent of the Finnish population lived in towns, only 21 per cent of workers were in industry and most of the industrial plants were small.^2^ Although the Finnish social morality regarding work was based on the agrarian value of work as a duty, problems, such as malingering, were coming under a new kind of scientific scrutiny when the war reparations forced Finland to leap into the world of industrial mass production.

In September 1944, Finland was obliged to pay $300,000,000 in gold to the Soviet Union, in the form of ships and machinery, within 6 years. Furthermore, under Soviet pressure, Finland had to refuse Marshall Plan aid despite losing a full tenth of its territory, relocating 420,000 evacuees from Karelia and planning the rehabilitation of disabled soldiers. The war reparations project was led by a tight cadre of industrial engineers who idealised Taylorism, but it was soon realised that the implementation of scientific management would require some harmony between labour, capital and welfare.^3^ It was in this context that a group of reformist physicians with English language skills found their niche in occupational medicine and began to stress ‘the supreme importance of good health as a contribution to social welfare and the national economy’.^4^

As a rule of thumb, physician and the first director of FIOH Leo Noro (1915–80) leaned on the joint declaration of the World Health Organization (WHO) and the International Labour Organisation (ILO) in defining modern health care as prevention, protection and risk management in striving for the optimum level of health:

Occupational health should aim at the promotion and maintenance of the highest degree of physical, mental and social well-being of workers in all occupations; the prevention amongst workers of departures from health caused by their working conditions; the protection of workers in their employment from risks resulting from factors adverse to health; the placing and maintenance of the worker in an occupational environment adapted to his physiological and psychological equipment and, to summarize: the adaptation of work to man and each man to his job.^5^

FIOH became a key hub for promoting the significance of social medicine, an emerging subfield of medicine that regarded health as a state of complete physical, mental and social well-being, not just the absence of disease.^6^ In practice, FIOH’s medical experts conducted evidence-based research in the field of occupational medicine, which was considered an integral part of social medicine. In so doing, they assumed a new, influential position in the society with an aim to disseminate novel medical knowledge to improve the overall quality of life of the working population.

While the broad context of this article is Finnish post-war occupational medicine, the empirical analysis offers insight into the development of work psychology in the post-war era.^7^ The idea for the article evolved from my extensive research in FIOH’s archives, where I focused on FIOH’s international correspondence during the war reparation years. The archival findings indicate that the Rockefeller Foundation was an important catalyst for the development of occupational medicine in Finland.^8^ After its establishment in 1948, WHO played an important supportive role with its projects and advisory services. In addition, FIOH participated in an active knowledge exchange, for instance, with the London School of Hygiene and Tropical Medicine, Columbia University School of Public Health, the American Medical Association (Council on Industrial Health), the British Medical Association and the Harvard School of Public Health.

Yet what primarily aroused my curiosity was the correspondence between FIOH and Roffey Park Rehabilitation Centre, which was established in West Sussex in 1943 by the National Council of the Rehabilitation of Industrial Workers. Many studies have investigated the role of the Tavistock Institute of Human Relations in the development of modern work psychology, but Roffey Park has been largely ignored.^9^ At the FIOH archives, however, I quickly discovered that there were no mentions of Tavistock in the immediate post-war period. Instead, there were several leaflets, letters and reports on study trips that described Roffey Park as an extraordinarily well-organised rehabilitation centre with impressive results in treating work-related neurosis.^10^ Once I started following these leads, I found myself in the middle of a multi-layered case study filled with a rich tapestry of events and a web of networks between medical communities.

This article has as many layers as the case study it investigates does. In the following section, I explain my theoretical framework and how it has facilitated my analysis of the archival findings. Then I present the context in which FIOH was established to provide a brief outline of its early years and core topics. Next, I elucidate the concept of *biological rationalisation*, which was often used in FIOH’s writings, by locating it in the broader transnational context of industrial medicine and the mental hygiene movement. This first part of the article is mainly based on my investigations of FIOH’s archives and close readings of texts written by FIOH’s experts to complement the larger picture.

The second section of this article explores the rehabilitative ethos at Roffey Park as well as the modern treatments administered there. I also investigate Roffey Park’s Training Department as a place where experts with different backgrounds could gather and discuss the reasons why people develop neurotic illnesses, the best approaches to treating them and how to prevent the spiral of workplace neurosis. Here, the analysis revolves around sources from the archives of the Wellcome Collection, publications related to Roffey Park and material from the FIOH archives.

In the final part of the article, I closely examine a report written by Roffey Park lecturer Dr Roger Francis Tredgold (1911–75) after his visit to Finland in 1950 as a WHO-appointed consultant. This section brings together the key issues investigated in the article by offering insights into the connections between FIOH, Roffey Park and WHO’s mental health programme. This case study therefore not only fills a research gap in the history of Finnish occupational health but also provides a new perspective on the role of transnational medical communities in disseminating medical knowledge.

## Medical Epistemic Communities

While the post-war period offered a new societal role for experts on the human mind, Finland was hardly an organised society steered by experts; rather, it was a volatile country that had been unprepared for the harsh war reparations. When hastily constructed correspondence courses intended to educate foremen on the war reparations industry were introduced in 1946, they included topics related to industrial psychology with an emphasis on mental health as a positive value. In these courses, however, the conformity of the worker manifested in stoicism and the will to work.^11^

The close connections between the state, industry and occupational medicine make it tempting to analyse the developments in work psychology in Foucauldian terms as a new instrument to control the social order. However, as Harry Yi-Jui Wu has noted in his research on WHO mental health programmes in the post-war era, it is unlikely that medicine was deliberately used for this purpose. Instead, the aim was ‘to address the burden of mental illness for individuals and communities’ and ‘to understand why certain people were particularly at risk for certain mental symptoms’.^12^ In this particular case, it is also important to recognise that the older generation of Finnish physicians considered the idea of FIOH’s Psychological Department ‘odd’. Physician and social democrat Oskari Reinikainen (1885–1969), who was Director-General of the State Medical Board, gave it his approval mainly because he did not find any reason to say no: ‘I really don’t understand why they need a Psychological Department, but because Noro and Oksala demand it, it is hard to disagree’.^13^

To highlight the importance of occupational health reforms, FIOH’s experts were keen to find reference points from other countries to show not only how things *could be* organised but how they *should be* organised. The UK was an important reference point to prove that Finland lagged behind other socially developed countries in research and the development of medical services—and indeed, many ideas of universal health care were not fully realised until public health reforms were enacted in the 1960s and 1970s.^14^ The archival material indicates that FIOH’s experts were already seeking spaces for collaboration with transnational medical communities in the 1940s to accelerate reforms towards universal health care. In fact, WHO was using Finland as a model country in occupational health recommendations for its member countries in 1957.^15^

To examine the knowledge exchange between FIOH and Roffey Park, the analysis utilises Peter Haas’ concept of an *epistemic community*, which roughly refers to professional networks with authority, similar values and worldviews, and policy-relevant expertise.^16^ In the analysis, FIOH and Roffey Park are both defined and scrutinised as *medical epistemic communities*, which were formed by experts from various subspecialities in medicine to concentrate on a problem of high societal concern and to offer practical solutions to absenteeism and maladjustment by scientific means.

Since the term epistemic community was coined, there has been significant academic discussion about the grounds on which a group of experts can be claimed to constitute an *epistemic* community. My inquiry follows Mai’a K. Davis Cross’ redefinition of the concept, according to which a strong, persuasive epistemic community must be internally cohesive. In other words, for a group of experts to be designated an epistemic community, they must share professional norms, a strong common culture and personal bonds.^17^ This definition applies to FIOH and Roffey Park, but instead of focusing narrowly on the internal cohesion within a single medical epistemic community, I seek a transnational perspective on the ways that medical epistemic communities exchange and disseminate knowledge despite having different resources, cultural differences and geopolitical tensions.

Consequently, my analysis takes inspiration from the concept of a *trading zone* that Peter Galison originally used to describe physicists and engineers working together to achieve the same outcome from different perspectives.^18^ While the archival material analysed in this article contains a source bias, and we cannot know what really happened in the meetings between FIOH’s and Roffey Park’s experts, the idea of a trading zone provides a theoretical framework for understanding how experts from different medical subspecialities can find ways to collaborate to reach similar outcomes.^19^ In practice, knowledge exchange was a prerequisite for FIOH to update the outdated German medical tradition with advanced theories and practices from English-speaking countries.

In these circumstances, FIOH’s experts took on the new role of mediators between the state, workers’ unions and employers’ associations with the aim of disseminating novel medical knowledge. Due to its persuasive yet unpolitical role, FIOH was a strong epistemic community with a socially recognised scientific authority and an ability to influence a wide variety of societal actors with conflicting interests.^20^ In the popularised health propaganda, the line between scientific and quasi-scientific knowledge was sometimes blurred, but the main driver of the institute was to produce evidence-based medical knowledge that would have a long-term impact beyond the war reparations industry.

## FIOH as a Reformist Hub

The Occupational Medicine Clinic was established in 1945 to study acute and chronic illnesses caused, for example, by exposure to carbon monoxide.^21^ Soon, it became evident that although the clinic was important, it was too small and impractical. Subsequently, Oskari Reinikainen proposed the construction of a research institute. Key actors in labour unions, industry, medicine and insurance companies supported the idea, and the Finnish Parliament approved the plan for the institute in 1946.^22^ Before FIOH was nationalised in 1978, it was an independent foundation supported by the government.

FIOH’s experts conducted mass X-ray surveys with mobile equipment in industrial plants throughout Finland in the years immediately after the war. The most frequent findings were tuberculous lesions, cardiac changes, lung cancer and pneumoconioses (silicosis and asbestosis).^23^ As rationalised and coordinated mass experiments are expensive, Noro used his personal contacts with medical professionals in the USA to forge connections with the Rockefeller Foundation. Noro’s connections, which were intensified during his Rockefeller-funded visit to the USA in 1947, were beneficial.^24^ The Rockefeller Foundation became FIOH’s key international supporter with a donation of $50,000 to purchase medical technology for the FIOH’s building, which was in the planning phase at that point.

When the institute was opened in 1951, it had a General Department for administration, a Medical Department, a Physiological Department, a Psychological Department, an Industrial Hygiene Department and a Counselling Section for Disabled Persons. Subsequently, the institute became a meeting place for experts from various medical and scientific sub-cultures. When German-educated psychologist Ohto Oksala (1905–84) began leading the Psychological Department, he was selected as leader of the new Department of Industrial Psychology at the Helsinki University of Technology as well. Thus, FIOH’s occupational psychology emerged in close connection with industrial engineering.^25^ Oksala’s nominations should therefore be contextualised within the broader development of institutionalised psychology in post-war Finland: departments of psychology were also founded at the Universities of Helsinki (1951), Tampere (1954) and Turku (1956).^26^ Oksala nonetheless had his own niche in the field of applied psychology with a focus on industrial psychology.

The post-war era was a heyday of medical statistics as well.^27^ FIOH relied on statistician Väinö Kannisto (1916–2002), who was a visiting scholar at the Johns Hopkins School of Hygiene and Public Health (1947–48) and the University of Pennsylvania (1948–49). The plan was unfortunately thwarted due to Kannisto’s anxiety about his possibilities for finding a decent job in Finland as a scholar specialising in statistics.^28^ After realising that WHO in Geneva offered better career prospects, Kannisto decided to pursue his career there, and he recommended that Noro find a way to educate a new statistician under the guidance of distinguished biostatistician Bradford Hill at the London School of Hygiene and Tropical Medicine.^29^ The Statistical Section was opened in 1953.

From today’s perspective, health care reforms were urgently needed. Despite developments in bacteriology and the introduction of antibiotics, tuberculosis remained stubbornly persistent, making the disease a main reason for lost work days in the 15–64 age cohort.^30^ Effective drugs entered the market between 1947 and 1951, but their high cost and limited availability posed a problem for many.^31^ In 1948, Noro used his personal connections to approach Standard Oil Company’s medical superintendent Robert C. Page with a personal matter: his wife was suffering from tuberculosis, and he was hoping to get a dose of the new streptomycin with instructions by airmail.^32^ The package arrived in Finland from the USA in a couple of weeks.^33^

Coronary heart disease posed another urgent medical problem, and physicians were alarmed that Finnish middle-aged men were among the leaders in coronary heart disease mortality.^34^ FIOH soon became a hub for investigations into the connections between hard manual labour, nutrition and cholesterol levels, which offered a starting point for the internationally acknowledged North Karelia Project launched in the early 1970s to monitor the risk factors of coronary mortality.^35^ Statistics were the standard of research at FIOH from the very beginning, and international comparisons of mortality, morbidity and health services were used to demonstrate the importance of preventive health care.

FIOH did not have an explicit mental health agenda. Instead, the institute utilised ideas from the mental hygiene movement that was supported by the Rockefeller Foundation in the inter-war period and by WHO in the late 1940s.^36^ In hindsight, Rockefeller’s post-war philanthropy was not unconditional, and it intertwined with exporting capitalism and investing heavily in European academic institutes to put science in the service of ‘“psychological warfare” against communist ideology’.^37^ Irrespective, the correspondence between FIOH and Rockefeller, first and foremost, centred on FIOH’s urgent need to respond to the needs of the war reparations industry. Noro nonetheless often used the USA as an example of a well-organised society with cutting-edge occupational medicine in his writings. In a magazine for industrial production managers in 1952, Noro shared the lesson he had learned from Robert C. Page: ‘Industry does not care about sick people or treating them. It cares about prevention: how to keep them healthy and get the best out of them’.^38^

## Biological Rationalisation

While FIOH’s experts moved beyond analysing the worker’s body as if it were a machine, they were advocates of Taylorism and referred to the working body explicitly as *the human machine* (ihmiskone). In their writings, FIOH researchers stressed the human side of rationalisation by distinguishing *technical rationalisation* and *biological rationalisation*. The former referred literally to the machines and raw materials, whereas the latter was an umbrella concept for *physiological rationalisation* and *psychological rationalisation.*^39^

As the concept of biological rationalisation reveals, the languages of biology, efficiency and nerves remained closely bound. In practice, investigating and rationalising the human nervous system was seen as the key to scientific and economic progress. Oksala’s key publication, *The Psychology of Work* (1948), for instance, compared the human soul to an electric motor. Like Frederick W. Taylor and his followers, Oksala converted the working subject into an object:

Technicians, those men, who have developed the electric motor, have utilised the laws of physics about electromagnetism …. The foremen and the ones who have developed the sciences of work have basically the same but much more difficult task ahead when they have to organise the ‘field forces’ in their workplace so that every worker’s soul—that is, their ‘compass needle’—settles in the same constellation with the forces within the social field so that the motor of their soul would start ‘rolling’—that is, working as efficiently as possible.^40^

Oksala’s book was a compilation of letters written for a correspondence school educating foremen in industry, but it remained the quintessential book on Finnish work psychology until the late 1960s. In the book, Oksala used German-born American social psychologist Kurt Lewin’s (1890–1947) field theory (topological psychology) to adopt concepts from physics, such as energy, tension, valence and vector (force), and elaborate psychodynamic theories of personality to promote ideas of mental hygiene at work.

The use of Lewin’s theories can be considered an example of the transnational hybridity in FIOH’s research and writings. Lewin was among the German Jewish émigrés who received assistance from the Rockefeller Foundation in the 1930s to continue his research in the USA. Among FIOH’s experts, Oksala was an exception because he did not speak English, but by adopting ideas from Lewin, he was able to connect the dynamic language of energy with Taylorism and early twentieth-century German theories on body types, temperament and character.^41^ As biological rationalisation was all about connecting somewhat conflicting theories and traditions, it had a strong neurological orientation, but it could also be used to explain why some workful people may experience nervous weakness or even break under the strain of work.

In *Psychology of Work*, Oksala categorised fatigue into three different types of maladies of energy: *asthenia* (nervous weakness), *tiredness* (transient exhaustion) and *hypotonia* (lack of willpower). These categories overlapped and were sometimes in conflict. As asthenia stood for nervous weakness, something that had even afflicted Charles Darwin in his later years due to excessive brain activity, Oksala’s attitude towards *some* weary workers was affirmative.^42^ Fatigued workers nonetheless posed a problem for productivity, and they increased the fear that work morale might be declining, which would pose a serious problem for the war reparations industry.

Although Finns were accustomed to working hard, the post-war reconstruction work was often undertaken in harsh conditions in forests and factories. It was not easy to find workers who would volunteer to perform hard manual labour in extreme conditions. Furthermore, concerns among the elite over war reparations remained abstract for the common people, whose concerns were mainly focused on everyday life under austere conditions.^43^ There were shortages of everything, and as noted by British journalist W.R. Mead, who visited Finland in 1946, the Finnish resettlement programme ‘encouraged the principle of self-help’.^44^ While this workful spirit gave the impression of tremendous activity, many struggled to make ends meet.

To raise the spirits of forestry workers, the state organised competitions for loggers to stress the importance of the wood industry in Finland’s reconstruction. These competitions offered a sudden possibility for FIOH to investigate the bio-psycho-social dimensions of heavy manual labour.^45^ After studies conducted in 1945 and 1951 on the anthropological measurements of champion lumberjacks revealed similarities between worker efficiency and athleticism, FIOH’s researchers concluded that ‘physiological changes resulting from work and sport are basically similar’.^46^ By following the path cleared by the Harvard Fatigue Laboratory (1927–47), a joint venture by the Harvard Business and Medical Schools, FIOH’s researchers became fascinated with the question of physiological and mental equilibrium.^47^ While the former formed an impetus for the field of Finnish sports medicine, the latter offered a theoretical ground to develop work psychology.

The Harvard Fatigue Laboratory was—much like FIOH—a hub for researchers devoted to interdisciplinary research focusing on human endurance. The key figures at the Fatigue Laboratory were industrial psychologist Elton Mayo (1880–1949), whose research later became an important part of Roffey Park’s curriculum, and Lawrence J. Henderson (1878–1942), a professor of biological chemistry at Harvard University. The laboratory hosted several international scholars, but not Finns, whose intellectual home at that time was Germany. FIOH scholars were nonetheless able to form close connections with the medical experts who had worked at the laboratory. One of these collaborators was physiologist Ancel Keys (1904–2004), with whom Martti J. Karvonen (1918–2009), head of the Physiological Department, worked closely in the field of cardiovascular physiology.^48^

In terms of knowledge exchange, the key question here is *why* a very particular research programme that was developed in the basement of Harvard Business School had an influence on post-war Finland. One explanation might lie in the reason why the Rockefeller-funded fatigue laboratory was established in the first place: the unrest of the early 1920s in industry.^49^ As Robin Wolfe Scheffler has noted, in contrast to previous industrial fatigue research, the researchers began investigating champion marathon runners and their staff members and uncovered that fatigue was, in fact, psychological, not physiological, in nature. After the laboratory was closed in 1947, business leaders began to embrace Mayo’s work, which asserted that fatigue rarely occurred under the more moderate strain of industrial work.^50^

In this regard, the research at the Harvard Fatigue Laboratory was close to FIOH’s theorisations about biological rationalisation. Although biological rationalisation was defined as more humane than the industrial rationalisation of the early 1920s, it did not challenge the idea of the working body as a human motor that could be modulated and optimised towards perfection and to combat, for instance, idleness and fatigue.^51^ Consequently, a moralistic tone framed FIOH’s studies on sickness absenteeism, which were in line with the societal expectation that ‘normal’ people should find work and hold down a job, and the unwillingness to work was a sign of maladjustment.^52^ In a study conducted from 1942 to 1949 comprising 210,000 workers from various industries, malingering was considered an alarming and rising trend:

In spite of the general improvement in national health the absenteeism referred to as temporary diseases and ‘sickness days’ has clearly increased in the last ten years. The writer [Noro] considers that the greater social security, the increased utilisation (= abuse) of advantage while sickness lasts, and the many new examinations and treatments of modern medicine, etc., cause increased short absences from work which pass as ‘sickness’. A new ‘social disease’—malingering—has also appeared which will be of the greatest importance to the national economy.^53^

As the aim of the Finnish post-war social policy was to extract the maximum labour from the human resources to the last drop, it was quite rational that workfare was applied even to disability policies.^54^ Although subtle stoicism was considered the ‘right’ way of dealing with nervousness and anxiety, lessons from Roffey Park persuaded FIOH to focus more on the impact of environmental stress on productivity and well-being.

## Roffey Park Rehabilitation Centre

The Second World War formed the backdrop for the emergence of Roffey Park Rehabilitation Centre. The neurosis centre, developed under wartime conditions of uncertainty, offered residential treatment for 120 patients for a period of six to eight weeks, including comprehensive physical and psychiatric investigations.^55^ The centre also aimed to provide expert knowledge of how to deal with diverse cases of *industrial neurosis*—a loosely defined condition resembling *war neurosis,* which was used during the First World War to discuss functional syndromes, such as shell shock, neurasthenia and soldier’s heart (a heart condition with no organic basis).^56^ As the warfare of World War Two was directed at civilians in unprecedented ways, it was noted that there was no differentiation between neuroses occurring in the civil population and among troops, although ‘terms such as “shell shock” or “soldier’s heart” should be avoided because of their unfavourable connotations’.^57^

The idea of Roffey Park was based on wartime findings indicating that the vicious circle of neurosis could be stopped. Yet there were disagreements between psychiatrists about whether wartime treatments of war neurosis had been effective. Aubrey Lewis (1900–75), who had been a clinical director at Mill Hill EMS Hospital during the war, was particularly pessimistic. Based on a study of 120 neurotic soldiers who had been discharged from the army and treated in neurosis centres, he noted in *The Lancet* in 1943 that those who were unemployed after their treatment had shown ‘hypochondriacal psychopathy’ since childhood: ‘they had been excessively concerned over their physical health … They had, to use the current phrase, been prone to “psychosomatic illness”’.^58^ Lewis’ pessimistic message was that instead of rest, therapy and drugs, these men needed work that was suitable for their personalities. Lewis also raised concerns about the untrustworthy diagnostics of neurosis and the risks of compensation neurosis.^59^

Psychiatrist Thomas Ling, soon to be the director of Roffey Park, disagreed with Lewis. In *The Lancet*, he responded to Lewis with the suggestion of establishing residential training centres under psychiatric control for the ‘minority’ who break down under the stress of life, because ‘apart from the humanitarian aspect, the nation would be the chief beneficiary from this development’. He then continued, ‘in the post-war years we shall need every skilled worker available and will be in no position to allow a substantial number to deteriorate in the way described by Dr. Lewis’.^60^ Consequently, the idea behind Roffey Park was related to the ‘pressing necessity of maintaining worker health to ensure productivity during the war and the immediate post-war period’.^61^

The rehabilitation centre was supposed to secure industrial production, but it also reduced the stigma of mental illness. Roffey Park’s promotional material conveyed the impression of neurosis as a moderate condition, separated from severe mental illnesses. In an article published in the *British Medical Journal,* the typical case at Roffey Park was described as presenting ‘a wide variety of symptomatology, *maladjustment* being the most important of them’.^62^ Yet maladjustment can mean anything from the failure to cope with the social environment to deep depression. These conceptual problems were raised in a confidential report written by the Roffey Park advisory panel appointed by the governors of St Thomas’ Hospital:

Up to 31st December, 1948, treatment had been provided for 4,150 patients who were selected with care to ensure that they were suitable cases to benefit from the facilities available. The type of case admitted has been referred to as ‘industrial’ neurosis, but this is a loose expression which has no medical significance and can mean anything beyond the fact that the patient has been engaged in some form of work.^63^

Roffey Park’s publications indicate that industrial neurosis was used as a quasi-medical term for a wide variety of symptoms, from fatigue and moodiness to depression and morbid anxiety.^64^ Although Roffey Park’s medical experts admitted that it was difficult to determine all the precipitating factors in neurotic illness, the promotional leaflets conveyed that there were promising effective treatments that would ‘cure’ the patients and enable them to satisfactorily settle back into working life.

A promotional leaflet marketing Roffey Park described six case examples of patients with ‘some typical problems’ treated at the institute. The first case, Albert G (28), was sent to Roffey Park due to backache, which had led him to receive workmen’s compensation for 8 months, after which he began suffering from anxiety. The second case, Margaret S (22), had poor health and was dealing with difficult situations at home with her mother and at her bombed workplace, all of which she was reacting to adversely. The third case, George D (38), had collapsed in the army in 1944. After being discharged, he returned to work, where he was depressed, apathetic and suffering from poor appetite. The fourth case, Robert W (28), had problems in settling into work after being a prisoner of war for 2 years in Germany. Case five, May B (26), suffered from malnutrition, insomnia and depression caused by overwork and an ‘irrational’ female supervisor. The last case, George D (36), was admitted to hospital as he was unable to work owing to a concussion and a serious arm injury sustained in a road accident, after which he feared going out alone. He had received ‘electrical treatment’, and when he was sent to Roffey Park, he was considered unemployable.^65^

At least some of the example cases described in the promotional leaflets indicate that industrial neurosis was occasionally used as an umbrella concept for traumatic war experiences, from being subjected to the Blitz to being a prisoner of war. When Roffey Park’s Training Department was opened in September 1947, it became a meeting place for industrial doctors, nurses, general practitioners, psychiatrists, social workers and trade union activists to discuss the psychological problems of adjustment and the ways neurotics could be ‘cured’ such that they could return to their duties.^66^ Supported by its wide network, Roffey Park’s contributions to occupational medicine were not so much scientific as they were social, educational and inspirational.

Roffey Park’s advisory panel affirmed the utmost importance of disseminating knowledge: ‘there is no doubt that the Training Department has made an important contribution to the lay teaching in industry’.^67^ This significance was presented numerically: by November 1948, 5-day and 2-day residential courses had been given to a total of 354 people, 27 of whom were foreign lay personnel and 39 foreign doctors.^68^ The credit for this educational work was given to Ling, with whom Noro had already had personal correspondence before he participated in the courses in 1948.^69^ Roffey Park’s significance was noted even by the Finnish state: when Dr Ling visited Finland in 1948, a presidential audience was organised for him.^70^ Yet the picture of the neurosis centre becomes much more complicated if we take a closer look at the treatments given for neurosis.

## Advanced Treatments at Roffey Park

A promotional leaflet described the treatments given at Roffey Park as ‘the most modern’.^71^ With their focus on a healing environment, the leaflets depicted Roffey Park as a sanatorium—something very different from a bleak mental institution. The building was located in the countryside and had a dance room, cosy common rooms, a library, a dining room and gyms for physical training. Meetings in these rooms were described as democratic and affirmative: ‘The friendly club atmosphere prevails in the dining hall. Making new friends is as valuable as rest and a change of occupation’.^72^ Like the sanatorium life in tuberculosis hospitals, possibilities to rest, eat and share thoughts with comrades in fate were supposed to create a feeling of therapeutic community.^73^

The promotional leaflets’ positive message about novel treatments, complemented by images depicting a warm and friendly atmosphere, was certainly attractive to a wide audience, from medical professionals to lay people. Notwithstanding the benign intentions, Roffey Park was, above all, a hospital offering full clinical, neurological and psychological investigations ‘to reverse the spiral of neurotic adjustment’.^74^ As a simple rest cure was considered useless for maintaining fitness at work, the premises were equipped with first-class workshops for active work therapy.^75^ As the rehabilitative ethos was to give patients self-confidence to resume performing their duties as soon as possible, the utilisation of work therapy is hardly a surprise. What is more striking is that biological treatments, such as electroconvulsive therapy, were in use as well.

Biological treatments were given at St Thomas’ Hospital, where Roffey Park’s advisor, psychiatrist William Sargant (1907–88), began working after the war. During the war, Sargant was certain that he had proved electroconvulsive therapy and insulin coma to be the right treatment for acute war neurosis.^76^ Sargant also recommended prefrontal leucotomy as a productive way of treating severe cases of chronic battle neurosis. In an article published in 1947, Sargant and C.M. Stewart presented a case report in which a 36-year-old man was recommended a leucotomy due to post-war fatigue, depression, mild paranoia (but not schizophrenia) and hypochondriac neurotic symptoms.^77^ According to *The Lancet,* some of Roffey Park’s patients were ‘cured’ with this risky treatment as well: ‘four were recommended for pre-frontal leucotomy and sent to hospital for the operation; they have done well, one of them making an exceptionally good recovery’.^78^

With the knowledge we have today, it is difficult to understand why these potentially dangerous treatments would have been used for treating ‘mild’ mental disorders. It is possible that the cases treated at Roffey Park were more severe than described in the leaflets or, before the medical revolution, these treatments were considered fast and cheap solutions to deal with the acute problem of overcrowding mental health care.^79^ To further contextualise this complicated issue, it is worth noting that during wartime, the ‘old conflict between the neurologist and psychiatrist’ had made ‘its appearance in England’.^80^ It seems that Roffey Park offered a meeting place for people and ideas from medical subspecialities that were distinct but close enough to share some activities.^81^ Exceptional times heighten the need to overcome internal disagreements in order to form a consensus, even if it means fusing conflicting theories.

It is equally important to recognise that the British war psychiatrists worked in tandem with the British Ministries of Information and Health. Although uncertainty was a normal state of wartime affairs, it was vital to convince the allies that at least some treatments for wartime neurosis had been found. Consequently, educational films, such as *Neuro Psychiatry* (1943), introduced a range of treatments, from insulin coma to social and occupational initiatives but emphasising the latter.^82^ In opposition to this, Sargant’s educational movie highlighted biological treatments as cost-effective and suitable for all kinds of neurotic patients—‘sailors, airmen, civilians’—in a full hospital.^83^ In the film, Sargant considered these promising biological treatments applicable: ‘All these methods can be used in the treatment of peace-time illness. But there is still much to learn. We are only at the beginning of developments in this field of research’.^84^

Before the development of neuroleptics (antipsychotics), biological treatments had their critics, but as they turned incurable diseases into treatable ones, they inspired optimism in medical professionals worldwide. In Finland, insulin coma and Cardiazol had been used in medical institutions to treat severe mental illnesses since the late 1930s, and in war psychiatry, they were used to get men back to the front line.^85^ In FIOH’s *Handbook for Industrial Hygiene and Occupational Medicine* (1951), Finnish psychiatrist Erkki Jokivartio, who had attended Roffey Park’s courses in 1948, welcomed biological treatments as promising examples of rehabilitation and resettlement:

Previously, those who were mentally ill were expected to take a coffin with them to the mental institution, which was their final station. But now, these so-called active treatments, such as insulin, Cardiazol and electroshock therapy, seem to bring most of the patients back to being respectable citizens who are fit for work.^86^

The biological treatments were presented in a positive light as a possibility for enabling work-shy neurotics to hold down a job. Otherwise, Finnish psychiatrists were not particularly interested in responding to the sufferings caused by the war. Instead, there was a strong tendency to consider neurotic reactions predominantly constitutionally determined with an inherited predisposition. Consequently, and quite obviously, psychotherapy was a marginal phenomenon in post-war Finland. From this perspective, the ground-breaking treatments given at Roffey Park were not biological treatments but something less physically intrusive—talk therapy.

Roffey Park’s psychiatrists promoted greater acceptance of expressing feelings and talking things through. The rehabilitation centre’s innovation was *sociatry*—methods for producing a co-operative atmosphere—‘a word that came into common usage during the war but is seldom heard to-day’.^87^ To build a friendly community, experts at Roffey Park drew ideas from wartime findings from the effort syndrome unit at the Mill Hill hospital. At the hospital, psychiatrist Maxwell Jones (1907–90) had advanced the concept of *therapeutic community* after realising that patients could deal better with their situation once the hospital hierarchy was made less rigid and they had the possibility to discuss the mechanisms of psychosomatic illnesses. In April 1947, with the financial assistance of several ministries, Jones opened an industrial neurosis unit for studying and treating chronic unemployed neurotics at Belmont Hospital. In the unit—comparable to Roffey Park—talk therapy was accompanied by physical exercise, occupational therapy and biological treatments.^88^

Talk therapy had its critics, especially due to the absence of statistical evidence, but it can be situated among the broader endeavours in a movement towards a more humane stance in dealing with distressing experiences. But was that really something new? The Rockefeller-funded mental health experts in industrial medicine, Elton Mayo in particular, had already fostered ideas about the sociology of the intimate and talking things through in the 1930s. During wartime, Mayo connected his previous theorisations on troublemakers directly to the wartime horrors: ‘if our social skills had advanced step by step with our technical skills, there would not have been another European war’.^89^ Loyal to his previous writings, Mayo considered socialists examples of unbalanced neurotics who could be cured with talk therapy. To prove this point, he presented the recovery of a man from the extreme left in a case study:

He discovered that his medical adviser was not at all interested in his political theories but was very much interested in the intimate details of his personal history. He made a good recovery and discovered, to his astonishment, that his former political views had vanished. He had been a mechanic, unable to keep his job although a good workman. After recovery he took a clerical job and held it; his attitude was no longer revolutionary.^90^

Mayo’s writings fed directly into the needs of industry, and they formed an important part of Roffey Park’s curriculum. It is questionable to what extent Roffey Park’s ideas differed from Rockefeller’s mental hygiene programmes aimed at making people mentally and physically fit for industrial work. A similar criticism can be levelled at psychiatrist Rodger Francis Tredgold’s book, *Human Relations in Modern Industry* (1949), based on his lectures at Roffey Park. The book, drawing on Mayo’s work, presented rather simplistic ideas about personnel selection and pejorative evaluations of accident-prone workers, social misfits and ‘destroyers’.^91^ The book nonetheless stressed the responsibility of medical practitioners, unionists, policymakers and the industrial elite to acquaint themselves with knowledge of workplace neurosis, share it and use it to resolve problems of maladjustment. Tredgold even dedicated his book to ‘those persons of sound mind who are interested in the welfare of their fellow workers’.^92^

While Roffey Park’s experts expressed naïve ideas about workplace democracy as conformism, they had some progressive ideas about recognising the early signs of anxiety disorders to ensure that they would be considered at work. Furthermore, the training offered medical professionals and lay people a possibility to reflect and discuss the various stressors in the post-war working environment. In the late 1940s, Roffey Park’s specialised treatment of neurosis was subsumed into general psychiatric provision in the British National Health Service (NHS).^93^ Similar ideas were nonetheless looming in WHO’s Mental Health Section, which was set up in 1949 with an aim to contribute to preventive mental health work together with governmental organisations.^94^ The motivations behind Roffey Park were certainly not altruistic, but they took into consideration the possibility that extreme stress and poor working conditions are psychosocial hazards.

## Disseminating Medical Knowledge

When WHO’s Mental Health Section initiated its activities, Tavistock consultant and Unilever’s Medical Officer Ronald Hargreaves (1908–62) was appointed to lead it. At this point, he turned his previous focus from military psychiatry to everyday stresses in modern society.^95^ Wartime attention to preventing mental disorders spilled over into the research initiatives at WHO, where mental disorders were increasingly framed as social, cultural and environmental.^96^ Unlike Roffey Park, WHO was not supposed to become a training department but a facilitator that would find suitable researchers to manage the projects or problems at hand to respond to each country’s needs.^97^ It was in this context that Roffey Park lecturer Tredgold was appointed to visit Finland in 1950 as a WHO consultant.^98^

It is likely that FIOH’s experts turned to the WHO due to the growing societal demand for mental health services, which, at this point, were clearly underdeveloped.^99^ Once Tredgold’s trip to Finland was agreed upon, Hargreaves and Noro decided that he would give lectures on the topics of industrial neurosis, rehabilitation and related services in the UK.^100^ During his visit, Tredgold familiarised himself with the key factories and (psychiatric) hospitals, where he noted the ‘unnatural’ split between the Hospital Board (beds) and the Ministry of Social Affairs (public health). After the visit, he submitted a report to WHO in which he was critical of Finnish psychiatry. When delivering the report, Norman D. Begg, an acting director at WHO’s Special Office for Europe, wrote to Noro: ‘as you will see this report is written in a personal strain and we do not propose to circulate it. However, I am sure you would wish to know what Dr. Tredgold has to say’.^101^

To summarise, Tredgold’s criticism considered the fact that Finnish physicians were more concerned with curing illnesses than with offering preventive health care. Mental health was therefore receiving little consideration:

The psychiatric lessons of the war do not seem to have been generally learnt; it is often said that there were few psychiatric soldier casualties, and little anxiety among civilians. But enquiry from a doctor in charge of a war neurosis centre gave me the impression that incidence and treatment of neurosis was much as in the British army; except that gross hysterical conditions replaced anxiety states. The pioneer ‘companies’ were formed of dullards.^102^

While Tredgold admitted that Finns had other urgent problems, such as 200,000 disabled people and high incidences of prematurity and infectious childhood diseases, he stated that in Finnish psychiatry, mental disorders were wrongly regarded as purely physical. Finns claimed that there were few psychiatric casualties and little anxiety among civilians, but ‘the war has left problems in its train, e.g. a period of “unsettledness”, an increase in alcoholism, and many disabled people’.^103^ Consequently, Tredgold recognised the pressing need for research into alcoholism due to ‘a high incidence of alcoholism, of criminal lunatics, and crime is often committed under the influence of alcohol’.^104^ The problem of alcohol consumption indeed became a matter of national interest when the Finnish Foundation for Alcohol Studies was established in 1950.

Tredgold’s criticism targeted outdated German (war) psychiatry. In the report, he stated bluntly that the elder generation of Finnish doctors was a strange ‘mixture of extreme social hospitality and intellectual apathy’.^105^ As the scientific language in Finnish medicine was German, and many well-educated Finns had poor English skills, it is likely that the visitor encountered language barriers. Despite taking this into consideration, it is obvious that Tredgold did not approve the idea that neurotics would be attention-seeking ‘war shakers’, who would benefit from their ‘abnormal psychological reactions’.^106^ With the knowledge we have today, at least some cases categorised as hysteric reactions in Finnish post-war and war psychiatry might have been diagnosed differently, possibly under the category of post-traumatic stress disorder or acute stress disorder.

Finnish psychiatrists were particularly reluctant to award pensions based on vague diagnostic categories such as war neurosis. The societal concern was that any kind of special treatment would lead to floods of claims for compensation and provide an excuse for malingering among work-shy labourers.^107^ While the Finnish ideal of work as duty did not differ much from Tredgold’s lessons at Roffey Park or the attempts to categorise and detect accident-prone, potentially neurotic workers, Tredgold made remarks on the high labour turnover, absenteeism and strikes in Finland which, according to him, many dismissed as political. Tredgold seems to have been referring to the Finnish elites’ anxieties about industrialisation and the unexpected popularity of left politics. The radical left party was banned from 1929 to 1944 due to anti-communist laws, but in the 1945 elections, the left parties were successful, raising the spirits of the working class.^108^ After this, trade union activities had new supporters, and Finland underwent an ongoing series of strikes, including the General Strike of 1956.

The fear that industrialisation could lure a new group of social misfits to the evolving cities was apparent in *The Handbook for Industrial Hygiene and Occupational Medicine* (1951). In a section on industrial psychiatry, psychiatrist Erkki Jokivartio wrote that ‘when people from the countryside move to industrial centres, many so-called schizophrenically restless, psychopathic individuals may come along’.^109^ While Roffey Park’s lessons about misfits fed directly into the Finnish industrial elite’s fears about the gloomy side of industrialised society, the training courses also encouraged listening to problem individuals with sympathy and tolerance. A qualified expert would then be able to recognise the ‘small fraction’ of ‘incurable ones’ and register them as disabled persons. After medical investigations, chronic troublemakers could be ‘withdrawn from general circulation in industry where they do nothing but harm. Others may respond to the special treatment available, enough to take full employment again’.^110^

At first sight, the difference between psychopaths and misfits may seem like a trivial detail, but it, in fact, exemplifies the difference between the German and British schools of thought. Most Finnish psychiatrists still relied on the German concept of psychopathy, whose origins lie in the late nineteenth-century doctrine of degeneration.^111^ Roffey Park’s take on industrial neurosis indicated that *anyone* may break down under extreme pressure. This new paradigm in mental health was promoted by WHO as well: the wartime lessons illuminated that stress among ordinary people is likely to be a costly burden to society.^112^ What is notable in the Finnish case is that the introduction of the new paradigm did not come solely from WHO but also from Tredgold, who was invited to visit Finland after active knowledge exchange between FIOH and Roffey Park.

It nonetheless took more than two decades for the emergence of radical socio-political consciousness to coincide with social policies and mental health care.^113^ In the post-war era, Finnish medical communities and social scientists accepted, for instance, racial hygiene as a ‘necessary’ precaution to control the birth rate among the unfit.^114^ In practice, eugenic policies increased in Finland due to the amendment to the 1935 Sterilisation Act in 1950, which stated that sterilisation could be carried out on eugenic, medical or social grounds until 1971.^115^ Finns, however, distinguished between ‘good’ (American) eugenics and ‘bad’ (German) eugenics as the former was expected to increase the vitality of the nation. An example of positive eugenic policy was mentioned in Tredgold’s report, and it considered the assimilation of the ‘pyknic and extroverted’ Karelians into the stern Lutheran Finnish society: ‘the Finns hope that the distribution of the 400,000 Karelian refugees all over Finland will be good eugenics’.^116^

In the final part of the five-page report, Tredgold concluded that Finns were in a difficult geographical and political position. They felt ‘cut off’, but ‘younger people are very alert to learn’ and therefore they ‘welcome every visitor’.^117^ Tredgold recommended that many Finns go abroad to enforce the connections to transnational medical communities. Not surprisingly, he recommended that Roffey Park would be a perfect meeting place for Anglo-Scandinavian scholars. With intellectual and financial support from WHO, an advanced course on human relations in industry for visitors from Scandinavia was subsequently organised in September 1951. In the leaflet, it was described as follows:

This course has been especially planned for Scandinavian Executives, Works Managers and Personnel Managers, Industrial Medical Officers and others concerned with problems of human relations in the light of current British practice. The course provides an examination of Industrial Relations and in particular the use and problems of non-financial incentives. There will also be a survey of recent research work and of application of psychology to industry. Discussion will form an integral part of the course.^118^

Language plays a key role in knowledge dissemination. Although Oksala was the leader of the Psychological Department, he was not able to participate in Roffey Park’s training due to his poor English skills.^119^ Instead, WHO granted scholarships to the younger generation of FIOH’s experts, and in the 1990s, psychologist Sauli Häkkinen reminisced about having learned lessons on human relations during a study trip 20 to 30 years before they became mainstream in Finland.^120^ In any case, Elton Mayo’s theories about teamwork and conformism, similar to those taught at Roffey Park, were added as a two-page appendix to the second edition of Oksala’s *The Psychology of Work* (1952).

## Conclusion

This article was intended to explore the knowledge exchange between medical epistemic communities—namely, FIOH and Roffey Park—and the impact of this collaboration on the development of Finnish work psychology. The analysis of this multi-layered case study suggests that FIOH’s reformists adopted ideas from Roffey Park by fitting them into Finnish post-war industrial medicine, which relied heavily on Taylorism, as exemplified in biological rationalisation. The reformist position gave FIOH the possibility of theorising psychological problems in industrial society anew, but these initiatives should be situated among broader transnational endeavours in the mental hygiene movement.

The establishment of FIOH was the first serious attempt to establish a research institute for investigating the bio-psycho-social requirements of work. FIOH’s experts introduced some progressive ideas for occupational health care, but the case study analysed in this article presents a complicated picture of the treatments of industrial neurosis. Industrial neurosis—like war neurosis—was a mosaic of the problems encountered in the wartime and post-war societies, making it a non-disease and a compilation of symptoms that could be recognised and classified in multiple ways depending on diverse interests. The concept of industrial neurosis enabled some doctors to rally around the view that neurotic conditions were due to heredity or weak nerves; simultaneously, it endorsed a positive view of the possibility of recovering from the various hardships encountered during and after the war.

As the Finnish social morality regarding work was stern, FIOH’s theorisations tended to emphasise neurobiological explanations with a moralistic tone. Biological rationalisation, for instance, conceptualised the human body as a dynamo that supplies nervous force, which in ideal conditions *should* turn into labour power. New ideas from Roffey Park likely persuaded FIOH’s experts to step outside the biomedical rationale and focus more on psychosocial hazards at work.^121^ In the 1950s, these new ideas were exemplified, for instance, in *manager’s disease*, a condition that appeared in those who had ‘burned their candle at both ends’.^122^ Although stress-related illnesses, including heart diseases, were approached with an individualised ethos, they were used as cautionary examples for building a road map for universal health care.

## Footnotes

1 Erkki Leppo, ‘Englannin, Sveitsin ja Pohjoismaiden teollisuuden terveydenhoito-oloista’, 11 (unpublished report, FIOH, 1948). Folder ‘Presentations and Manuscripts 1949’, FIOH archives, Helsinki, Finland (henceforth FIOH archives).

2 Only 758 establishments had more than 75 workers; 2,118 had between 10 and 75, and more than 65,000 plants had less than 10 employees. Leo Noro, ‘Occupational Health in Finland’, *Occupational Medicine*, 1948, *5*, 607–13, 607.

3 Pauli Kettunen, ‘The Society of Virtuous Circles’, in Pauli Kettunen and Hanna Eskola, eds, *Models, Modernity and the Myrdals* (University of Helsinki: Renvall Institute Publications, 1997), 153–73.

4 Leo Noro, *Occupational Medical Foundation 1945–1955 / Institute of Occupational Health 1951–1955* (Helsinki: Valtioneuvoston Kirjapaino, 1956), 17.

5 Noro, *Occupational Medical Foundation 1945–1955*, 5.

6 William Hobson, ‘What Is Social Medicine?’, *British Medical Journal*, 1949, 4619, 125–30, 127.

7 The umbrella term work psychology was used in FIOH’s publications written in English; therefore, I used it throughout the article. The Psychological Department was, for instance, described as a unit concentrated on matters of work psychology and clinical-psychological studies in co-operation with the medical unit. Noro, *Occupational Medical Foundation 1945–1955*, 6.

8 Rockefeller financially and intellectually supported the nursing education in Finland during the inter-war era. Ann Yrjälä, *Public Health and Rockefeller Wealth. Alliance Strategies in the Early Formation of Finnish Public Health Nursing* (doctoral dissertation, Åbo Akademi, 2005).

9 Finnish scholars mainly approach the formation of the human relations movement and the therapeutic society via Nikolas Rose’s work on governmentality. Rose mentions Roffey Park once, but I have not seen any mentions in the Finnish research literature. Nikolas Rose, *Governing the Soul: The Shaping of the Private Self*, 2nd edn (London: Free Association Books, 1999), 51.

10 These positive impressions were also reported in the Finnish key medical journal. Leo Noro, ‘Havaintoja teollisuus- ja sosiaalilääketieteestä Englannissa, Ranskassa ja Belgiassa’, *Duodecim*, 1949, *65*, 155–72.

11 Mona Mannevuo, *Ihmiskone töissä. Sotienjälkeinen Suomi tehokkuutta tavoittelemassa* (Helsinki: Gaudeamus, 2020).

12 Harry Yi-Jui Wu, *Mad by the Millions: Mental Disorders and the Early Years of the World Health Organization* (Cambridge: The MIT Press, 2021), 34.

13 Sauli Häkkinen, *Työterveyslaitoksen psykologian osasto 1950–1970* (Helsinki: Työterveyslaitos, 1993), 1.

14 Minna Harjula, ‘Health Citizenship and Access to Health Services: Finland 1900–2000’, *Social History of Medicine,* 2016, *29*, 573–89, 575.

15 Leo Noro, *Työterveyslaitos. Tausta ja kehitys vuoteen 1978* (Helsinki: Työterveyslaitos, 1979), 184.

16 Peter M. Haas, ‘Introduction: Epistemic Communities and International Policy Coordination’, *International Organisation,* 1992, *46*, 1–35.

17 Mai’a K. Davis Cross, ‘Rethinking Epistemic Communities Twenty Years Later’, *Review of International Studies*, 2013, *39*, 137–60.

18 Peter Galison, *Image and Logic: A Material Culture of Microphysics* (Chicago: The University of Chicago Press, 1997), 784.

19 Peter Galison, ‘Trading Zone: Coordinating Action and Belief’, in Mario Biagioli, ed, *The Science Studies Reader* (London: Routledge, 1998), 137–60, 145–47.

20 Cross, ‘Rethinking Epistemic Communities’, 159.

21 During the war, coal gas was used as fuel due to a shortage of gasoline. Tens of thousands of chauffeurs, engineers and workers were exposed to carbon monoxide. Noro, ‘Occupational Health in Finland’, 611.

22 FIOH’s history is not fully covered here, but it had an administration that included, for example, key government bodies, insurance institutions, the Faculty of Medicine at the University of Helsinki, the Finnish Institute of Technology, the National Pensions Institute, the Central Federation of Employers, the Federation of Finnish Trade Unions and the City of Helsinki. A Detailed History of FIOH: Noro, *Työterveyslaitos. Tausta ja kehitys vuoteen 1978*; Alpo Väänänen, *Työn jäljet. Työterveyslaitos 1945–1995* (Helsinki: Työterveyslaitos, 1995); Eino Ketola, *Majakka ja luotsi. Työterveyslaitos 1945–2015* (Helsinki: Työterveyslaitos, 2015).

23 Noro, *Occupational Medical Foundation*, 20–21.

24 L. Noro to R.C. Page, 11 September 1947, Folder ‘International Correspondence 1945–1947’, FIOH archives.

25 Petteri Pietikäinen, ‘Autonomy, Theory and “Applied” versus “Basic”. Work Psychology and Its Search for Identity in Finland, ca. 1945–2000’, in Sven Hroar Klempe and Roger Smith, eds, *Centrality of History for Theory Construction in Psychology* (New York: Springer, 2016), 187–207, 198.

26 Petteri Pietikäinen, ‘Finland’, in David B. Baker, ed, *The Oxford Handbook of the History of Psychology: Global Perspectives* (Oxford: Oxford University Press, 2012), 211–27, 221.

27 Harry M. Marks, *The Progress of Experiment*: *Science and Therapeutic Reform in the United States, 1900–1990* (Cambridge: Cambridge University Press, 1997), 148–55.

28 V. Kannisto to L. Noro, 23 January 1948, folder ‘Leo Noro’s Correspondence 1947–1951’, FIOH archives.

29 In spring 1948, Kannisto decided to take up a temporary position as a statistical expert at WHO on the project Wastage of Human Life. V. Kannisto to L. Noro, 5 May 1948, folder ‘Collegial Body 1950–1955’, FIOH archives; V. Kannisto to L. Noro, 19 October 1949, Folder ‘Collegial Body 1950–1955’, FIOH archives.

30 Leo Noro, ‘Työvoimaan kohdistuvan terveydenhoitotyön sosiaalinen ja taloudellinen tausta’, in Leo Noro, ed, *Teollisuushygienian ja työlääketieteen käsikirja* (Helsinki: Otava, 1951), 18–34, 29.

31 Heini Hakosalo, ‘The Ill(s) of the Nation: The Experience of Tuberculosis in Finland from the 1920s to the 1970s’, in Ville Kivimäki, Sami Suodenjoki and Tanja Vahtikari, eds, *Lived Nation as the History of Experiences and Emotions in Finland, 1800–2000* (Cham: Palgrave Macmillan, 2021), 241–66, 249.

32 L. Noro to R.C. Page, 5 May 1948, Folder ‘Leo Noro Correspondence 1947–1951’, FIOH archives.

33 R.C. Page to L. Noro, 7 June 1948, Folder ‘Leo Noro Correspondence 1947–1951’, FIOH archives.

34 Väinö Kannisto, ‘Miksi Suomen miehet kuolevat ennenaikaisesti?’, *Duodecim*, 1951, *67*, 1108–12.

35 Mikko Jauho, ‘Becoming the North Karelia Project: The Shaping of an Iconic Community Health Intervention in Finland (1970–1977)’, *Social History of Medicine*, 2021, *34*, 1212–35.

36 WHO’s Expert Committee on Mental Health considered mental hygiene important in industry because absences from work were often due to mental disorders. WHO, *WHO and Mental Health* (Geneva: World Health Organization, 1962), 15. On Rockefeller’s mental hygiene programme, see Mathew Thomson, ‘Mental Hygiene as an International Movement’, in Paul Weindling, ed, *International Health Organisations and Movements, 1918–1939* (Cambridge: Cambridge University Press, 1995), 283–304.

37 Olivier Zunz, *Philanthropy in America*: *A History* (Princeton: Princeton University Press, 2012), 146–51.

38 Leo Noro, ‘Terveys on valttia’, *Tehostaja*, 1952, *6*, 40–48, 46. Noro also published a popularised book in Finnish titled *Uncle Sam Looks after His Worker’s Health* (1949).

39 Ohto Oksala, *Työn psykologia (kirjeopetusta varten)* (Helsinki: Tietomies, 1948), Letter No. 1, pp. 6–8. The book is a compilation of 12 letters written in 1947 and 1948, but as I have used the whole book in this research, the year 1948 is referenced throughout the paper.

40 Oksala, *Työn psykologia*, Letter No. 1, p. 4.

41 Pietikäinen, ‘Autonomy, Theory and “Applied” versus “Basic”’, 192.

42 Oksala, *Työn psykologia,* Letter No. 3, p. 6.

43 Henrik Meinander, *Suomi 1944. Sota, yhteiskunta, tunnemaisema* (Helsinki: Siltala, 2009), 351–60.

44 W.R. Mead, ‘Reconstruction in Finland’, *The Norseman*, 1947, *6*, 397–406, 402.

45 Martti J. Karvonen, *Työterveyslaitoksen fysiologinen osasto 1950–1965* (Helsinki: Työterveyslaitos, 1991), 11–12; Olavi Eränkö and Martti Karvonen, ‘Body Type of Finnish Champion Lumberjacks’, *American Journal of Physical Anthropology*, 1955, *16*, 331–43. Today, this journal is known as *American Journal of Biological Anthropology*.

46 Noro, *Occupational Medical Foundation 1945-1955*, 22-23.

47 Jason Oakes, ‘Alliances in Human Biology: The Harvard Committee on Industrial Physiology, 1929–1939’, *Journal of the History of Biology*, 2015, *48*, 365–90; Robin Wolfe Scheffler, ‘The Power of Exercise and the Exercise of Power: The Harvard Fatigue Laboratory, Distance Running, and the Disappearance of Work, 1919–1947’, *Journal of the History of Biology*, 2015, *48*, 391–423.

48 Martti J. Karvonen, *Työterveyslaitoksen fysiologinen osasto 1950–1965,* 40; Jauho, ‘Becoming the North Karelia Project’, 1216–17.

49 Robin Wolfe Scheffler, ‘The Fate of a Progressive Science: The Harvard Fatigue Laboratory, Athletes, the Science of Work and the Politics of Reform’, *Endeavour*, 2011, *35*, 48–54, 49.

50
*Ibid.*, 52.

51 On the idea of the working body as a human motor, see Anson Rabinbach, *The Human Motor: Energy, Fatigue, and the Origins of Modernity* (New York: University of California Press, 1990).

52 Katariina Parhi and Petteri Pietikäinen, ‘Socialising the Anti-Social: Psychopathy, Psychiatry and Social Engineering in Finland, 1945–1968’, *Social History of Medicine,* 2017, *30*, 637–60, 641.

53 Leo Noro, ‘Työntekijäin sairauspäivistä Suomessa’, *Duodecim,* 1949, *65*, 953–73, 973.

54 Heli Leppälä, ‘Duty to Entitlement: Work and Citizenship in the Finnish Post-War Disability Policy, early 1940s to 1970’, *Social History of Medicine*, 2014, *27*, 144–64; Heli Leppälä, ‘Welfare or Workfare? The Principle of Activation in the Finnish Post-War Disability Policy, Early 1940s to Late 1980s’, *Journal of Social History*, 2016, *49*, 959–81.

55 Thomas Ling and V. W. Wilson, ‘A Survey of Occupational Problems in a Neurosis Centre’, *British Medical Journal*, 1952, *4783*, 558–60, 558.

56 Edgar Jones and Simon Wessely, *Shell Shock to PTSD: Military Psychiatry from 1900 to the Gulf War* (Hove: Psychology Press, 2005), 206.

57 William H. Dunn, ‘War Neuroses’, *Psychological Bulletin,* 1941, *38*, 497–504, 497.

58 Aubrey Lewis, ‘Social Effects of Neurosis’, *The Lancet*, 1943, *241*, 167–70, 167.

59 Edgar Jones, ‘*Neuro Psychiatry 1943*: The Role of Documentary Film in the Dissemination of Medical Knowledge and Promotion of the U.K. Psychiatric Profession’, *Journal of the History of Medicine and Allied Sciences*, 2012, *69*, 294–323, 304.

60 Thomas Ling, ‘The Neurotic Ex-soldier’ [Letter to the Editor], *The Lancet*, 1943, *241*, 348.

61 Jill Kirby, *Feeling the Strain: A Cultural History of Stress in Twentieth-Century Britain* (Manchester: Manchester University Press, 2019), 124.

62 Thomas Ling and V.W. Wilson, ‘A Survey of Occupational Problems in a Neurosis Centre’, 559. The article was based on a survey of 842 cases treated at Roffey Park in 1949–50. In terms of clinical disease, the survey showed 32 per cent depressive states, 47 per cent anxiety states and the remainder mixed states.

63 Report of the Advisory Panel appointed by the Governors of St Thomas’ Hospital in collaboration with Roffey Park Rehabilitation Centre, 1949, 2. William Walters Sargant Correspondence, PP/WWS/B/2/7, Wellcome Collection. Available at: https://wellcomecollection.org/works/j3fanwbr.

64 Anonym., ‘A Rest from Industry’, *The Lancet*, 1945, *246*, 607–608; Kirby, *Feeling the Strain*, 119.

65 Roffey Park Institute Limited*, Health and Human Relations in Industry*: *The Courses at Roffey Park*, 1947, 3–4, Wellcome Collection, General Collection 3359; see also Kirby, *Feeling the Strain,* 2019, 121–22.

66 Roger Francis Tredgold, *Human Relations in Modern Industry* (London: Gerald Duckworth, 1949), 177–78.

67 Report of the Advisory Panel, 2.

68
*Ibid.*, 2.

69 L. Noro to T. M. Ling, 10 September 1947, folder ‘International Correspondence 1945–1947 I’, FIOH archives; Noro to Ling, 7 June 1947, Folder ‘Documents 1945–1955’, FIOH archives.

70 Leo Noro to Alli Paasikivi [President’s wife], 9 February 1948, folder ‘Documents 1945–1955’. FIOH archives. In the letter, Noro wrote, ‘Dr. Ling is the Head of the Famous Roffey Park Rehabilitation Centre and a well-known expert in rehabilitation and industrial hygiene’.

71 Roffey Park*, Health and Human Relations in Industry*, 5–6.

72 Roffey Park Rehabilitation Centre, *Roffey Park Rehabilitation Centre: A Record for Two Years’ Progress*, 1944–1946 (London: National Council for the Rehabilitation of Industrial Workers, 1946), 11. Wellcome Collection, General Collection, WB320.

73 On patients’ experiences of tuberculosis hospitals, see Hakosalo, *The Ill(s) of Nation*, 263.

74 Ling and Wilson, ‘A Survey of Occupational Problems in a Neurosis Centre’, 558.

75 Roffey Park*, Health and Human Relations in Industry*, 5–6; Roffey Park Rehabilitation Centre, *A Record for Two Years’ Progress*, 7–8. On the history of British occupational therapy, see John Hall, ‘From Work and Occupation to Occupational Therapy: The Policies of Professionalisation in English Mental Hospitals from 1919 to 1959’, in Ernst Waltraud, ed, *Work, Psychiatry and Society, c. 1750–2015* (Manchester: Manchester University Press, 2016), 314–33, 320–22.

76 William Sargant and Eliot Slater, ‘Acute War Neuroses’, *The Lancet*, 1940, *236*, 1–2; William Sargant, ‘Physical Treatment of Acute War Neuroses’, *British Medical Journal,* 1942, *4271*, 574–76.

77 William Sargant and C. M. Stewart, ‘Chronic Battle Neurosis Treated with Leucotomy’, *British Medical Journal,* 1947, *4534*, 866–69.

78 Anonym., ‘A Rest from Industry’, 608.

79 See also Petteri Pietikäinen, *Madness: A History* (London: Routledge, 2015), 262–64.

80 Dunn, ‘War Neuroses’, 501.

81 Galison, ‘Trading Zone’, 146.

82 Jones, ‘*Neuro Psychiatry 1943*’, 314.

83 William Sargant, *The Treatment of War Neurosis*, 1942, National Film & Television Archive, Wellcome Collection; see also Ben Shephard, *A War of Nerves: Soldiers and Psychiatrists in the Twentieth Century* (Cambridge: Harvard University Press, 2000), 205–10.

84 Sargant, *The Treatment of War Neurosis*, 1942, Wellcome Collection.

85 In extensive research on Finnish war psychiatry, Ville Kivimäki concluded that these treatments were sometimes used as a form of punishment. Ville Kivimäki, *Murtuneet mielet. Taistelu suomalaissotilaiden hermoista, 1939–1945* (Helsinki: Otava, 2013), 383.

86 Erkki Jokivartio, ‘Näkökohtia teollisuuslaitosten psykiatrisesta huollosta’, in Leo Noro, ed, *Teollisuushygienian ja työlääketieteen käsikirja* (Helsinki: Otava, 1951), 314–32, 316. Evidence on Jokivartio’s attendance at Roffey Park’s courses, Noro to Ling, 3 August 1948, folder ‘Documents 1945–1955’, FIOH archives.

87 Ling and Wilson, ‘Survey of Occupational Problems in a Neurosis Centre’, 558. The writers drew the concept from Henry Harris, a social psychologist who was responsible for personnel selection in the British army. Henry Harris, *The Group Approach to Leadership-Testing* (London: Routledge & Kegan Paul, 1949).

88 Edward Shorter, *A History of Psychiatry*: *From the Era of the Asylum to the Age of Prozac* (New York: John Wiley, 1997), 232–34.

89 Elton Mayo, *The Social Problems of an Industrial Civilization* (Boston: Harvard University, 1945), 23.

90
*Ibid.,* 26–27.

91 Tredgold, *Human Relations in Modern* Industry, 161–63; Kirby, *Feeling the Strain*, 123–24. The word ‘destroyer’ was originally used by Elton Mayo.

92 Tredgold, *Human Relations in Modern Industry*, 5.

93 Kirby, *Feeling the Strain*, 125.

94 John Rawlings Rees, ‘By Way of Introduction’, *International Social Science Journal*, 1959, *11*, 7–13, 9.

95 On the role of Hargreaves in British military psychiatry, see Shephard, *War of Nerves,* 188–89.

96 Wu, *Mad by the Millions*, 30–34. See also Rose, *Governing the Soul*, 82.

97 Wu, *Mad by the Millions*, 34–38.

98 Oskari Reinikainen to Norman Begg, 24 February 1950, Folder ‘Foreign Correspondence, 1950–1951 II’. FIOH archives. The visit was mentioned in WHO’s publication focused on the developments in preventive mental health care: ‘a WHO consultant visited Scandinavia in 1950 to discuss mental hygiene problems arising in industry’. WHO, *WHO and Mental Health*, 15.

99 Pietikäinen, ‘Finland’, 222.

100 Leo Noro to Ronald Hargreaves, 29 June 1950, folder ‘Foreign Correspondence, 1950–1951 II’. FIOH archives.

101 Norman Begg to Leo Noro, 24 November 1950, folder ‘Foreign Correspondence, 1950–1951 II’, FIOH archives.

102 Roger F. Tredgold, ‘Report to Dr G. R. Hargreaves on visit to Finland’, 3 (unpublished report, FIOH, 1950). Folder ‘Foreign Correspondence 1950–1951 II’, FIOH archives.

103
*Ibid.*, 4.

104
*Ibid.*, 3.

105
*Ibid.*, 2.

106 On ‘war shakers’ and German military discipline in World War II, see Shepard, *War of Nerves,* 302–303.

107 In a similar vein, during the war, psychiatrists were reluctant to discharge any patients who had had nervous breakdowns or to agree to any compensation for war neurosis. Kivimäki, *Murtuneet mielet*, 373–75.

108 In the 1945 elections, the Social Democratic Party gained 25.08 per cent of the votes, and the more radical Finnish People’s Democratic League got 23.47 per cent of the votes.

109 Jokivartio, ‘Näkökohtia teollisuuslaitosten psykiatrisesta huollosta’, 316.

110 Tredgold, *Human Relations in Modern Industry,* 163.

111 Psychopathy was used in post-war Finland as a reference to ‘maladjusted’ individuals whose behaviour was considered grossly deviant or anti-social. Parhi and Pietikäinen, ‘Socialising the Anti-social’, 638–42.

112 Wu, *Mad by the Millions*, 48.

113 Pietikäinen, ‘Finland’, 223.

114 Heikki Waris, *Suomalaisen yhteiskunnan rakenne* (Helsinki: Otava, 1948), 39.

115 Marjatta Hietanen, ‘From Race Hygiene to Sterilization in Finland’, in Gunnar Broberg and Nils Roll-Hansen, eds, *Eugenics and the Welfare State: Sterilization Policy in Denmark, Sweden, Norway and Finland* (East Lansing: Michigan State University Press, 2005), 195–258, 239.

116 Tredgold, Report to Dr G. R. Hargreaves, 3. In research published in 1952, funded by the Rockefeller Foundation, it was stated that this adjustment process went smoothly. Recent research has, however, shown that Orthodox Karelians had to assimilate their culture and habits to the Lutheran social order. Heli Kananen, ‘The Adaptation of an Ethnic Minority in Finland in the 1940s and 1950s: Orthodox Displaced Persons and the Lutheran Indigenous Population’, *Scandinavian Journal of History*, 2013, *38*, 111–32.

117 Tredgold, Report to Dr G. R. Hargreaves, 4.

118 Advanced Course on Human Relations in Industry, 1951, Folder ‘Foreign Correspondence, 1952–1953’. FIOH archives.

119 Leo Noro to Ronald Hargreaves, 29 September 1950, Folder ‘Foreign Correspondence, 1950–1951 II’. FIOH archives; Hargreaves to Noro, 10 October 1950, Folder ‘Foreign Correspondence, 1950–1951 II’. FIOH archives.

120 Häkkinen, *Työterveyslaitoksen psykologian osasto*, 1993, 6.

121 See also Eve-Riina Hyrkäs, ‘Psychosomatic Pain? The Meanings of Musculoskeletal Affliction in Finnish Medicine, ca. 1950–2000’, *European Journal for the History of Medicine and Health*, 2021, *78*, 128–54.

122 Leo Noro, *‘Johtajantauti’ – Nykyajan tärkein ammattitauti* (Helsinki: Salaman kirjapaino, 1955), 1.

